# Epigenetic evidence of an Ac/Dc axis by VPA and SAHA

**DOI:** 10.1186/s13148-021-01050-4

**Published:** 2021-03-20

**Authors:** Sebastian Lunke, Scott Maxwell, Ishant Khurana, Harikrishnan K.N., Jun Okabe, Keith Al-Hasani, Assam El-Osta

**Affiliations:** 1 Baker Heart and Diabetes Institute, Melbourne, VIC 3004 Australia; 2 Epigenetics in Human Health and Disease Laboratory, Central Clinical School, Monash University, Melbourne, VIC 3004 Australia; 3 Department of Diabetes, Central Clinical School, Monash University, Melbourne, VIC 3004 Australia; 4 Department of Clinical Pathology, The University of Melbourne, Parkville, VIC 3010 Australia; 5 Department of Medicine and Therapeutics, The Chinese University of Hong Kong, Shatin, NT, Hong Kong SAR; 6 Hong Kong Institute of Diabetes and Obesity, Prince of Wales Hospital, The Chinese University of Hong Kong, 3/F Lui Che Woo Clinical Sciences Building, 30-32 Ngan Shing Street, Sha Tin, Hong Kong SAR; 7 Li Ka Shing Institute of Health Sciences, The Chinese University of Hong Kong, Shatin, NT, Hong Kong SAR; 8 Biomedical Laboratory Science, Department of Technology, Faculty of Health, University College Copenhagen, Copenhagen, Denmark

**Keywords:** Anti-epileptic drug, VPA, Chromatin, Acetylation/deacetylation (Ac/Dc), HDAC inhibitor

## Abstract

**Background:**

Valproic acid (VPA) is one of the most commonly used anti-epileptic drugs with pharmacological actions on GABA and blocking voltage-gated ion channels. VPA also inhibits histone deacetylase (HDAC) activity. Suberoylanilide hydroxamic acid is also a member of a larger class of compounds that inhibit HDACs. At the time of this article, there are 123 active international clinical trials for VPA (also known as valproate, convulex, divalproex, and depakote) and SAHA (vorinostat, zolinza). While it is well known that VPA and SAHA influence the accumulation of acetylated lysine residues on histones, their true epigenetic complexity remains poorly understood.

**Results:**

Primary human cells were exposed to VPA and SAHA to understand the extent of histone acetylation (H3K9/14ac) using chromatin immunoprecipitation followed by sequencing (ChIP-seq). Because histone acetylation is often associated with modification of lysine methylation, we also examined H3K4me3 and H3K9me3. To assess the influence of the HDAC inhibitors on gene expression, we used RNA sequencing (RNA-seq). ChIP-seq reveals a distribution of histone modifications that is robust and more broadly regulated than previously anticipated by VPA and SAHA. Histone acetylation is a characteristic of the pharmacological inhibitors that influenced gene expression. Surprisingly, we observed histone deacetylation by VPA stimulation is a predominant signature following SAHA exposure and thus defines an acetylation/deacetylation (Ac/Dc) axis. ChIP-seq reveals regionalisation of histone acetylation by VPA and broader deacetylation by SAHA. Independent experiments confirm H3K9/14 deacetylation of NFκB target genes by SAHA.

**Conclusions:**

The results provide an important framework for understanding the Ac/Dc axis by highlighting a broader complexity of histone modifications by the most established and efficacious anti-epileptic medication in this class, VPA and comparison with the broad spectrum HDAC inhibitor, SAHA.

**Supplementary Information:**

The online version contains supplementary material available at 10.1186/s13148-021-01050-4.

## Introduction

Histones in chromatin are covalently modified to provide a direct and precise mechanism to regulate gene expression [1]. The control of transcription involves core machinery and chromatin-modifying factors that serve as a functional linkage between histone deacetylase (HDAC) and histone acetyltransferase (HAT) enzymes. Indeed, histone lysine acetylation and deacetylation are controlled by a HAT/HDAC-axis, and the addition of acetyl groups to the ε-amino group of lysine residues by HATs and the removal of acetyl groups were catalysed by HDAC enzymes, respectively. Since histone acetylation is a mark for gene activation, studies analysing genome-wide mapping of HATs and HDACs on chromatin by immunoprecipitation combined with sequencing have reported dramatic histone acetylation changes conferred by HDAC inhibition [2].

HDAC enzymes in higher eukaryotes are classified by sequence homology to yeast proteins and comprise four groups all of which are zinc-dependent amidohydrolases except for the sirtuins [3]. Class I members include HDAC1, HDAC2, HDAC3 and HDAC8 and are predominantly localised in the nuclear compartment with ubiquitous tissue distribution. Class IIa members include the isoforms HDAC4, HDAC5, HDAC7 and HDAC9 localised in the nucleus and cytoplasm and distributed in the heart, skeletal muscle and brain, whereas class IIb members HDAC6 and HDAC10 are mostly cytoplasmic. Class III family members (SIRT1-7) are sirtuins that are dependent on nicotinamide adenine dinucleotide (NAD) for catalytic activity. Class IV contains HDAC11, which is the least homologous to yeast prototypes. Furthermore, the catalytic activity of class I, II and IV HDACs is inhibited by pharmacological actions of trichostatin A (TSA) and has a zinc-dependent active site. In contrast, class III sirtuins are inhibited by nicotinamide but not TSA.

HDAC inhibitors compromise structurally diverse compounds, and TSA was the first natural hydroxamate to inhibit catalytic activity [4]. Indeed, other hydroxamic acids such as vorinostat (zolinza, suberanilohydroxamic acid, SAHA) are classified as pan-HDAC inhibitors and inhibit the catalytic activity of classes I, II and IV [5]. SAHA is the first potent HDAC inhibitor to be approved for clinical use by the US Food and Drug Administration [6, 7]. The aliphatic acids are relatively weak inhibitors (millimolar concentrations) of class I and IIa HDACs and include compounds such as valproic acid [8–10]. VPA has a long history as an anticonvulsant with broad use in epilepsy and clinical neuroscience [11]. VPA was first approved by the FDA in 1978 under the tradename Depakene (Divalproex Sodium, Epilim, Convulex) and entered the World Health Organization’s core list of essential medicines for basic health care. Total prescriptions for the neuroprotective Divalproex oral tablet in the USA were 5,945,500 and primarily used in psychiatric conditions and as an anti-epileptic agent. The number of completed clinical trials registered by ClinicalTrials.gov is currently 447 public and private studies involving VPA and SAHA.

The most direct mechanism by which HDACs function to control acetylation levels are on transcribed genes, and this has traditionally been studied by pharmacological inhibition of HDAC activity. Chromatin immunoprecipitation combined with sequencing (ChIP-seq) has revolutionised our understanding of the epigenome and clearly shown that HDAC inhibition elevates histone acetylation levels on genes poised for activation [2]. More recent studies have questioned the extent of the Ac/Dc (acetylation/deacetylation) axis immediately following HDAC inhibition to reveal some surprising results [12]. Indeed, while data in vascular cells suggest hyperacetylation of lysine residues on histones as determined by ChIP-seq underlies transcriptional competence of active and poised genes, the contribution of histone deacetylation is by far the predominant signature in response to TSA. Genome-wide mapping of soluble lysine residues of histones in chromatin clearly shows pharmacological HDAC inhibition causes broad deacetylation in the heart [12, 13].

With the exception of cardiac and vascular cells, the histone world remains largely uncharted with regard to the physiological function of HDAC inhibitors, and deconvoluting the complexity of the acetylation/deacetylation-axis remains challenging. First, histone acetylation is often assessed by protein blotting, which complicates interpreting genome-wide patterns. For example, studies from our laboratory show hyperacetylation as a measure of total histone content is by no means informative of genomic location [8–10, 12, 14–21]. While the conventional view of elevated histone acetylation is the paradigmatic consequence of HDAC inhibition, this is likely to explain only a fraction of gene targets [22]. Second, genome-wide datasets from non-malignant cells are limited [15]. These considerations suggest that even though most genes are predicted targets of histone acetylation in response to HDAC inhibition, only a fraction of these interactions focus on active gene targets, while little is known of the influence on histone deacetylation [23]. This is supported by genome-wide mapping of HATs and HDACs in active, primed and inactive genes [2]. With mounting data predicting therapeutic benefit by restoring the lysine acetylation state, there remains a paucity in deacetylation substrates that medicinal chemistry has identified. Thus, the development of next-generation HDAC inhibitors faces significant challenges understanding the regulatory roles of HDACs in the genome [24].

In this study, we assessed the pattern of histone modifications, specifically, H3K9/14ac, H3K4me3 and H3K9me3 by ChIP-seq in primary human cells stimulated with the anticonvulsant drug VPA, which is used primarily to lessen the severity and frequency of seizures [25, 26]. We also performed independent validation experiments using the HDAC inhibitor, SAHA. Surprisingly, we found the effectiveness of VPA and SAHA to influence gene expression changes was not only associated with histone hyperacetylation, but also reduced histone acetylation (or deacetylation). This study revealed commonalities previously thought to be limited to cardiac cells: [1] VPA confers histone acetylation and deacetylation that was independently validated by using SAHA and is a feature of HDAC inhibition observed by genome-wide mapping techniques, [2] diverse changes conferred by HDAC inhibition for H3K4me3 and H3K9me3 associate with altered gene activities, and [3] HAT/HDAC-axis correlate with expression patterns that converge to regulate NFκB-dependent pathways.

## Materials and methods

### VPA and SAHA stimulation of fibroblast cells

Growth medium was decanted, and cells were exposed to Mock (vehicle), 5 mM VPA or 2 µM SAHA prepared in DMEM for 16 h. Concentrations of the pharmacological inhibitors VPA and SAHA demonstrating elevated histone acetylation signals and physiological relevance were derived from previous studies by our group [8–10, 12, 14–21]. ChIP-Seq and RNA-Seq were performed using protocols described previously by our group from five experiments for the untreated control, VPA and SAHA stimulated cells [12, 27].

### Chromatin immunoprecipitation (ChIP)

Approximately 12 × 10^6^ fibroblast cells were grown in four 150-mm cell culture dishes and to near confluency before VPA and SAHA stimulation. Media were decanted, and cells were incubated on the dish for 5 min with 20 ml ChIP fixation buffer (prepared fresh, 37% formaldehyde in PBS) with mild agitation on a platform mixer. ChIP fixation buffer was decanted and immediately replaced with 20 ml ChIP quenching buffer (Glycine prepared fresh in PBS), followed by 10-min incubation with mild agitation. After decanting the quenching buffer, cells were rinsed twice in ice-cold PBS and harvested by scraping in 5-ml ice-cold PBS. Cells were pelleted by centrifugation for 3 min at 300 × *g*, and supernatant was discarded. Cells were transferred into 1.5-ml safe-lock microcentrifuge tubes (Eppendorf) using 1-ml ice-cold PBS and pelleted for 10 min at 1000 × *g*, 4 °C. Cell pellets were resuspended in 300 µl of ChIP SDS-Lysis buffer at room temperature and sonicated for 30 min at high power at 4 °C using a Bioruptor (Diagenode). Samples were centrifuged for 10 min at 13.000 × *g*, 4 °C to remove insoluble chromatin. Soluble chromatin was transferred into a fresh 1.5-ml microcentrifuge tubes ready for the immunoprecipitation reaction.

DynaBeads Protein A magnetic beads (Invitrogen) (40 µl per ChIP reaction) were washed twice in 1 ml ChIP Dilution Buffer and resuspended. Up to 50 µl of soluble chromatin solution was reconstituted with 20 µl of pre-washed magnetic beads and 500 µl of ChIP Dilution Buffer and incubated for 2 h on a rotating wheel at 4 °C for pre-clearing. An equal aliquot of soluble chromatin was transferred to a separate 1.5-ml safe-lock microcentrifuge tube and kept at 4 °C to serve as input control sample at a later time. In parallel, 20 µl of pre-washed beads was incubated with 4 μg of ChIP grade antibody (H3K9/14ac 06-599 Sigma-Aldrich, H3K4me3 39,159 Active Motif and H3K9me3 8898 Abcam) in a total volume of 500 µl ChIP Dilution Buffer for 2 h, 4 °C, on a rotating wheel. Cleared soluble chromatin was incubated overnight on a rotating wheel at 4 °C, and the magnetic beads were washed consecutively with 1 ml of ChIP Low Salt Buffer, ChIP High Salt Buffer, ChIP LiCl Buffer, ChIP TE buffer and ChIP TE buffer containing 0.01% SDS using a rotation wheel at 4 °C for 5 min. To reverse cross-links, beads were incubated in 100 µl ChIP Elution Buffer including 2.5 µl proteinase K for 2 h at 62 °C in a thermo mixer (Eppendorf) at 1400 rpm. Similarly, the input control samples were combined with 100 µl ChIP Elution Buffer including 2.5 µl proteinase K and incubated alongside the ChIP samples. Input and ChIP samples were combined with 150 µl phenol/chloroform/isoamylalcohol and vortexed. Samples were centrifuged at 16.000 × *g* for 10 min at room temperature. The aqueous phase (~ 100 µl) was transferred to fresh 1.5-ml microcentrifuge tubes and DNA-purified using MinElute DNA extraction columns (Qiagen) according to manufacturer’s instructions. DNA concentration was determined using a Qubit QuantIt DNA quantitation kit (Invitrogen) according to the manufacturer’s instructions. Samples were stored at − 20 °C or used directly in next-generation sequencing library preparation.

### RNA isolation

Total RNA was isolated from cells grown to full confluency in 50-mm cell culture dishes using TRIzol reagent (Invitrogen) according to manufacturer’s instructions. Approximately 1 × 10^6^ cells were washed once with 5 ml PBS. TRIzol (Invitrogen) was added directly to cells, vortexed for 20 s and incubated at RT for 5 min. 200 µl chloroform was added to each tube and vortexed for 10 s. The sample was incubated at RT for a further 3 min and centrifuged for 15 min at 13,200 rpm (4 °C) to separate the aqueous and organic phases. The upper, aqueous phase containing the RNA was transferred to a new 1.5-ml microcentrifuge tube and placed on ice to precipitate with ice-cold isopropanol for 15 min. Precipitated RNA was pelleted by centrifugation for 10 min at 13,200 rpm (4 °C) and washed in 200 µl ice-cold 75% ethanol. The pellet was resuspended in ice-cold nuclease-free water and subject to RNase-Free DNase Set (Qiagen, cat. no. 79254) according to the manufacturer’s instructions. RNA was prepared using RNeasy Mini Kit RNA Cleanup protocol (Qiagen), and the concentration was determined using a Qubit QuantIt RNA quantitation kit (Invitrogen). Samples were stored at -80 °C or used directly in next-generation sequencing library preparation.

### Library preparation for massive parallel sequencing

In brief, 5 ng of purified and fragmented DNA derived from ChIP was ligated to Illumina paired-end adapters and size-separated using agarose gel electrophoresis to remove unligated adapters. A size range of 200–300 bp was excised from the gel, and the library DNA was recovered. Libraries were amplified by PCR using Illumina paired-end primers. For mRNA-Seq, 10 µg of total RNA derived from cells was purified using magnetic oligo-dT beads. Purified mRNA was chemically fragmented and converted to double-strand cDNA. Illumina paired-end adapters were ligated to the cDNA. The library was size-separated using agarose gel electrophoresis to remove unligated adapters. A size range between 175 and 225 bp was excised after electrophoresis. Libraries were amplified by PCR using Illumina paired-end primers. Integrity and concentration were determined by analysing a portion of the library using a MCE-202 MultiNA Microchip Electrophoresis System (Shimadzu) and DNA-500 separation Kit (Shimadzu) according to manufacturer’s instructions. Libraries were resuspended in Tris–EDTA buffer for Illumina sequencing.

### Computational analyses

Data derived from Illumina paired-end sequencing (DNA) and single read sequencing (RNA) was demultiplexed using bcltofastq (Illumina) according to manufacturer’s instructions. All files were filtered for unaligned reads before further processing. For DNA (ChIP-Seq)-derived data, reads were aligned to the human genome (hg19) using Burrows Wheeler Aligner (BWA) [28]. Data were further analysed using Model-based Analysis of ChIP-Seq (MACS) peak-finding algorithm [29] to identify regions of enrichment against an input control, as well as differential enrichment of treated vs untreated (Mock) control samples. SeqMonk was used to visualise aligned ChIP-Seq reads (RRID:SCR_001913). Peak calling was performed using model-based analysis of ChIP-Seq (MACS) peak-finding algorithm [29]. Intersection analysis was performed for genomic features using the BEDTools collection of scripts for the histone modifications H3K9/14ac, H3K4me3 and H3K9me3 [30]. Distribution of histone modifications at genomic features including CpG Islands and defined windows around the transcription start site (TSS) was counted and stored in an epigenomic quantitation database (EQDB) using scripts in perl, python and R as well as sqlite3 database. Detailed analysis of H3K9/14ac, H3K4me3 and H3K9me3 was performed by assessing the distribution of reads over 2500-bp region upstream and downstream of annotated RefSeq TSS using SeqMonk trend plot feature and R. Cumulative read counts were adjusted for library size. Transcription factor binding site (TFBS) analysis was performed for genomic sequences 5-kb region of the TSS. Fasta sequences were extracted from the hg19 genome using Galaxy [31]. TFBS analysis for non-redundant JASPAR binding motifs [32] was performed using Clover [33]. Sequence data for TSSs of RefSeq genes were used in the analyses.

RNA sequencing data were aligned to the human genome (hg19) using RNA-Aligner Mapsplice [34] and differential expression analysis using edgeR [35]. Cufflinks was used for de novo transcriptome assembly, fragment length normalisation, annotation of the transcripts and differential expression analysis for RNA-Seq using the RefSeq (hg19) mRNA annotation set [36]. Custom scripts combined with MACS-derived statistics were used to determine genomic features associated with histone modification and transcript abundance. Empirical cumulative distribution function (ECDF) was used for correlating peak distance with transcript abundance. The results were plotted using R, visualising the relative cumulative fraction of differential peaks for histone modifications relative to TSSs associated with changes in gene expression. BEDTools was used to develop scripts to assess histone acetylation shoulders associated with differential gene expression (30). Gene ontology and pathway analysis for the results of differential expression analysis were performed using the GeneGo MetaCore data analysis suite (www.genego.com). All statistical analyses and data visualisations were performed using RStudio (http://www.rstudio.com/). Significance is defined as an adjusted *P*-value of < 0.05 with a coefficient of variance for the target transcript of < 0.5 (between replicates).

### Quantitative PCR validation of histone modification by ChIP and RNA transcript abundance

Real-time PCR was performed on a 7500 Fast Real-Time PCR System (Applied Biosystems). The reaction mix contained 10 µl 2 × FastStart Universal SYBR Green Master including Rox (Roche), 5 pmol each of forward and reverse primer, 5 µl TE buffer and 4 µl of the respective sample. The PCR conditions were as follows: 95 °C for 10 min, 40 cycles, 95 °C for 3 s and 60 °C for 30 s. Fluorescence data for each cycle were collected after the 60 °C elongation step for the analysis of product concentration. Following amplification, a melting curve analysis was performed according to the instrument protocol. Results were analysed using real-time PCR 7500 software v2.0.4 (Applied Biosystems). Oligonucleotides used in qPCR for ChIP validation were prepared in nuclease-free water at 10 pmol concentration. Oligonucleotide pairs used for the following genes were as follows: NFKB1 fwd GGCAGCGACCCTACCTCCCG and NFKB1 rev CGCTCCGGTGGCGAAACCTC; NFKB2 fwd GCCCCGCCTCCCCTTGGTAT and NFKB2 rev GGCGGGTGAGATCCGGTGGA; RelA fwd ACAGCCCGGATGGGACGACT and RelA rev CCCACAGCCGATGAGAGCCG; RelB fwd CTGCCCAACCCCTCCTGAGC and RelB rev GGGAAAACCCCCACGCGTCA. Validation of transcript abundance for NF-kB family genes was performed using TaqMan Fast Advanced Master Mix (Applied Biosystems) and transcript-specific TaqMan assays (Applied Biosystems) according to manufacturer’s instruction. Expression levels of 18S were used as endogenous control. Applied Biosystems assays are as follows: NF-kB1 (nuclear factor kappa B subunit 1) TaqMan assay ID Hs00765730_m1 with FAM fluorescence label; NF-kB2 (nuclear factor kappa B subunit 2) TaqMan assay ID Hs00174517_m1 with FAM fluorescence label; RelA (RELA proto-oncogene, NF-kB subunit, p65) TaqMan assay ID Hs00153294_m1 with FAM fluorescence label; RelB (RELB proto-oncogene, NF-kB subunit) TaqMan assay ID Hs00232399_m1 with FAM fluorescence label and 18S (rRNA endogenous control) TaqMan assay ID 4319413E with VIC fluorescence label. qPCR assays were performed in three independent experiments.

### Datasets availability

Available from the corresponding author on reasonable request.

## Results

### Genome-wide mapping reveal diverse changes in histone modification by VPA and SAHA

**Fig. 1:**
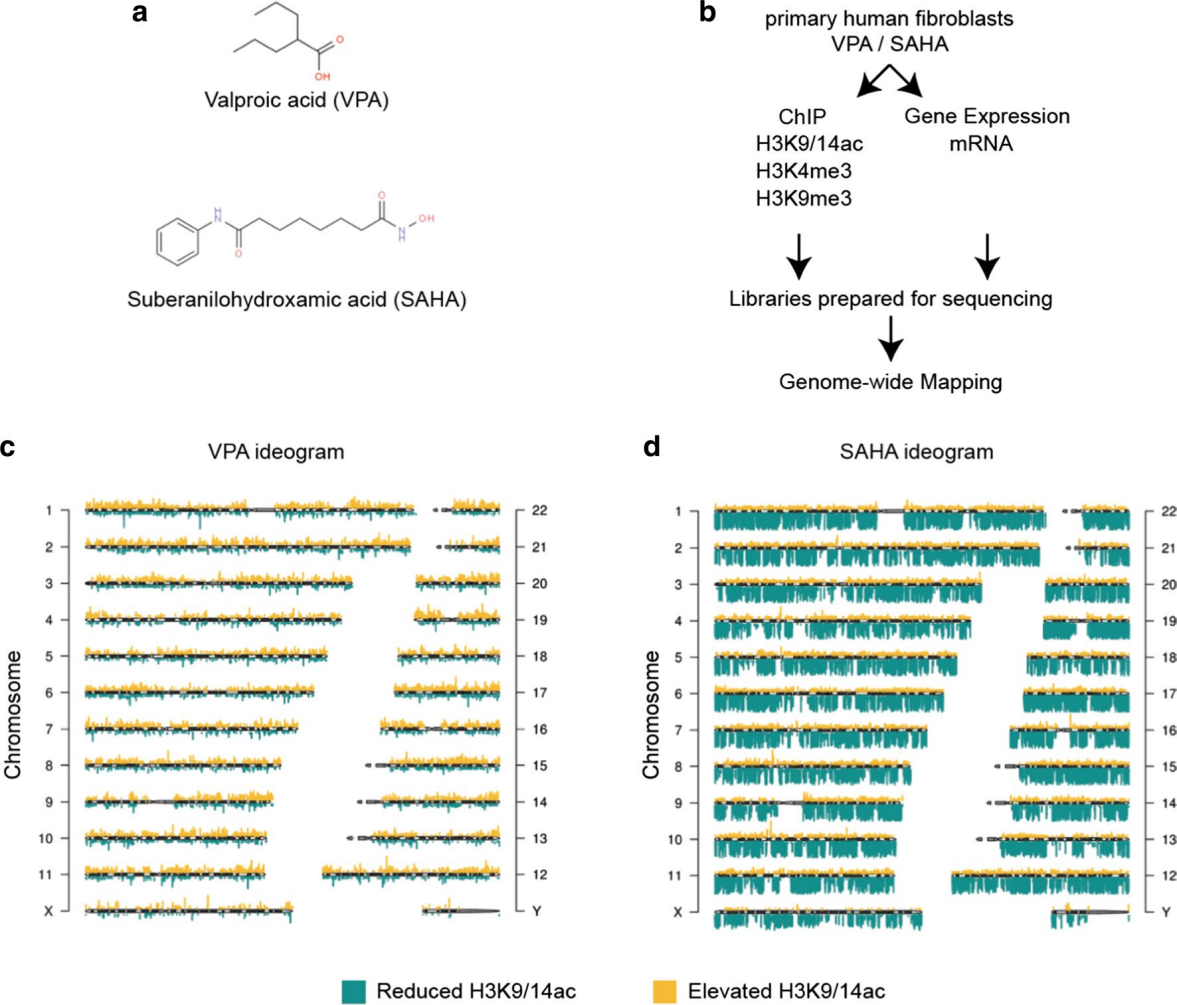
Pharmacological inhibition by VPA and SAHA caused broad acetylation and deacetylation patterns. **a** Chemical structures of VPA and SAHA. **b** Plan of experiments and analyses performed using chromatin immunoprecipitation (ChIP) using antibodies that specifically recognise H3K9/14ac, H3K4me3 and H3K9me3 and gene expression using massive parallel sequencing and mapping. ChIP-Seq was performed from five experiments and RNA-Seq from three experiments for the untreated control and cells exposed to VPA and SAHA. **c** ChIP followed by sequencing (ChIP-seq) shows MACS (*Bonferroni-adjusted P* < *0.05*) derived bipartite acetylation (elevated H3K9/14ac in yellow) and deacetylation peaks (reduced H3K9/14ac in green) in cells stimulated with VPA. **d** ChIP-seq shows MACS (*Bonferroni-adjusted P* < *0.05*) derived acetylation (elevated H3K9/14ac in yellow) and deacetylation peaks (reduced H3K9/14ac in green) in cells stimulated with SAHA

Outside of the brain, the fibroblast is the most numerous cell type in the body. To build the first instalment of an anti-epileptic atlas of the epigenome, we used ChIP-Seq to map histone modifications in primary human fibroblasts. First, we exposed cells with derivatives of the aliphatic acids, VPA and SAHA as shown in Fig. 1a. Chromatin immunoprecipitations were performed using antibodies that specifically recognise acetylated histone H3 (H3K9/14ac) and trimethylation of histone H3 (H3K4me3 and H3K9me3), respectively. Libraries were prepared for massive parallel sequencing in cells stimulated with VPA and SAHA (Fig. 1b).

The distribution of H3K9/14ac (schematically shown on the chromosome ideogram) is dynamic in VPA stimulated cells (Fig. 1c). Broad increases in H3K9/14 acetylation levels (yellow-histone acetylation) are observed and coincide with reduced acetylation (green-histone deacetylation) following VPA exposure. While regions of increased acetylation are apparent, a dramatic correlation is observed for histone deacetylation by SAHA (Fig. 1d). In VPA-exposed cells, the levels of increased and decreased acetylation are balanced, but deacetylation is more pronounced in cells exposed to SAHA.

**Fig. 2:**
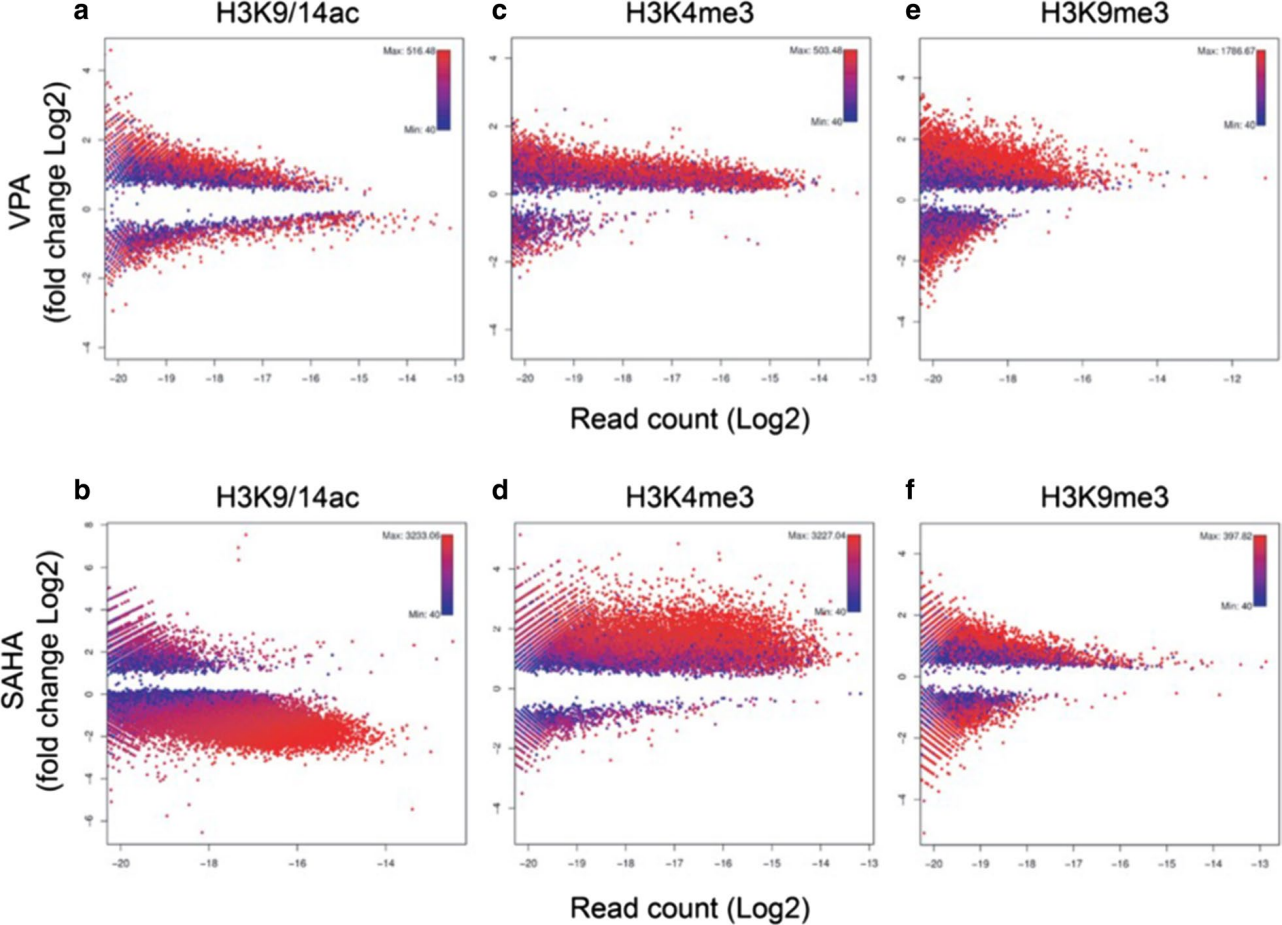
Genome-wide distribution of chromatin modifications shows significant changes in histone modification by VPA and SAHA. Distribution plots show the read count (log2 read abundance) compared with fold change (log2FC) of differential regions determined by edgeR, comparing VPA and SAHA stimulated against unstimulated human fibroblast cells (*Bonferroni-adjusted P* < *0.05*). ChIP-seq regions subject to significant change are shown in red and non-significant change shown in blue for VPA- and SAHA-stimulated cells. ChIP-seq distribution plots are shown for VPA **a** H3K9/14ac, **c** H3K4me3 and **e** H3K9me3 and SAHA **b** H3K9/14ac, **d** H3K4me3 and **f** H3K9me3. All peaks considered significant by MACS (*P* < 10^–4^) are included. Higher scores represent stronger changes

Because acetylation-mediated gene expression is often associated with modification of other histone lysine residues, we also assessed H3K4me3 and H3K9me3. We plotted regions subject to significant changes in red (adjusted *P* < 0.05) and non-significant changes shown in blue for cells exposed to VPA and SAHA. Increased histone acetylation was observed and enriched at sites with low acetylation indices before pharmacological HDAC inhibition. Histone deacetylation was also observed with VPA exposure (Fig. 2a) and most obvious in response to SAHA (Fig. 2b). These results suggest the epigenome is subject to widespread histone deacetylation at genomic regions previously acetylated. In comparison with H3K9/14ac, we detected a small number of regions identified with decreased H3K4me3 following HDAC inhibition with gains in H3K4me3 more readily identified for VPA (Fig. 2c) and SAHA (Fig. 2d). Changes to H3K9me3 were moderate after SAHA exposure than those observed for H3K9/14ac and H3K4me3. Interestingly, VPA and SAHA exposure elevated H3K9me3 (Fig. 2e and 2f). We did not observe significant changes in H3K27me3 and DNA methylation in cells stimulated with the HDAC inhibitors (data not shown).

### VPA and SAHA cause distinct changes to gene expression

**Fig. 3:**
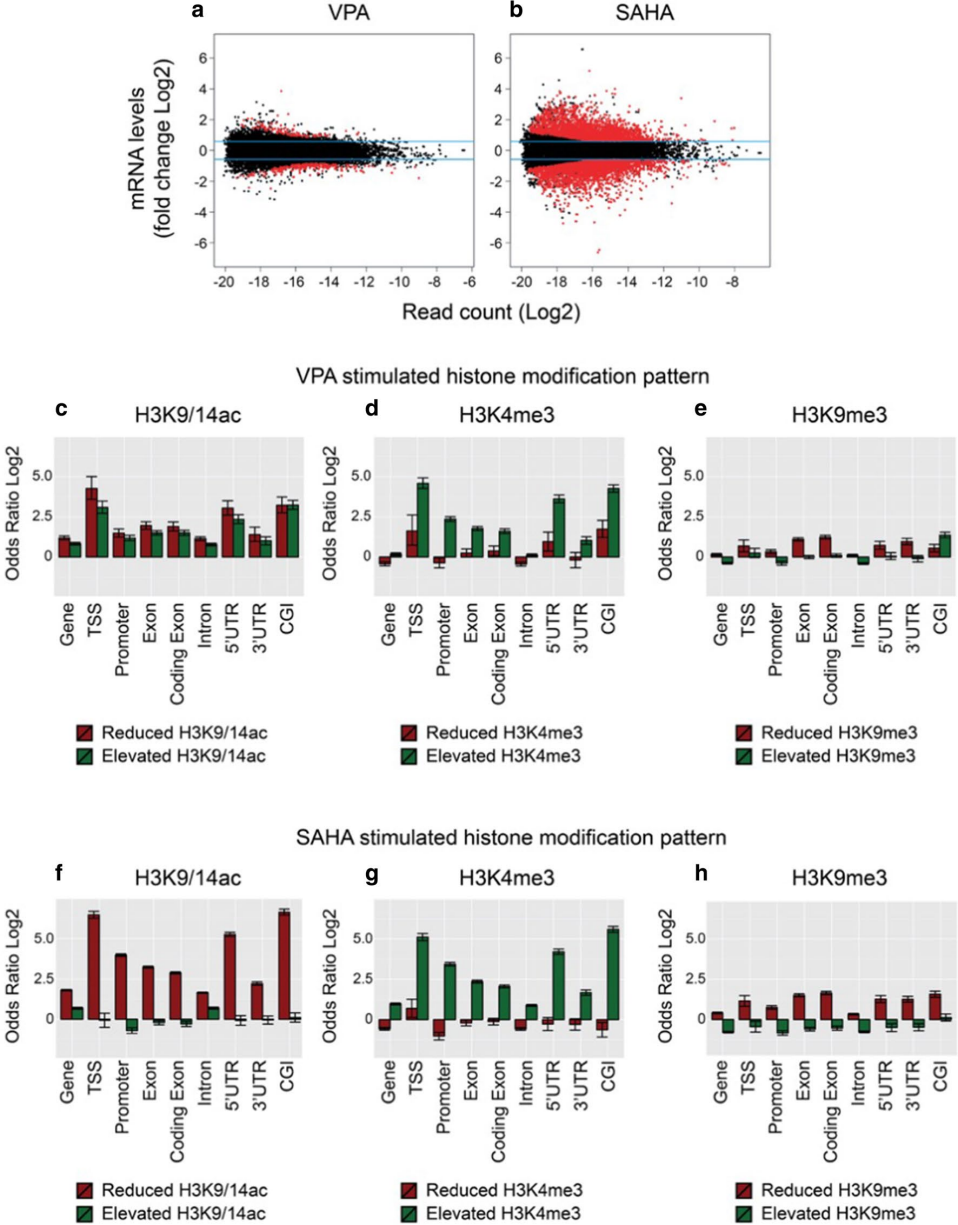
Contribution of genomic features exposes differential chromatin modifications using VPA and SAHA. **a**, **b** Distribution plots show the read count (log2 read abundance determined by edgeR) compared with fold change (log2FC). Transcripts subject to significant change are shown in red (adjusted *P* < 0.05) and non-significant change shown in black for cells exposed to VPA and SAHA. For each transcript, the fold change between RNA-seq data sets from stimulated and unstimulated cells was plotted against the combined read count for the corresponding gene. Significant differences in mRNA levels are shown in red and non-significant change shown in black for VPA- and SAHA-stimulated cells. The overlap of genomic features and regions of differential histone modification in VPA and SAHA exposed cells are also shown. ChIP-seq peaks were analysed for genomic features using odds ratio testing compared to ChIP input data selected as reference. VPA-stimulated genomic features are shown for **c** H3K9/14ac, **d** H3K4me3 and **e** H3K9me3. SAHA-stimulated genomic features are shown for **f** H3K9/14ac, **g** H3K4me3 and **h** H3K9me3

To define the relationship between chromatin modification and gene expression, libraries from RNA-Seq were analysed in cells stimulated with VPA and SAHA. The analysis of the transcriptional response to VPA identified 520 genes that were differentially regulated (Fig. 3a, *P* < 0.05, max. Coefficient of variation ≤ 0.5). We observed strong regulation of lipid metabolism pathways by VPA, which is consistent with the chemical structure as a short-chain fatty acid (Additional file 1: Table S1). This observation suggests that a sizeable fraction of differential gene expression may be driven by the metabolism of its short-chain fatty acid structure. In contrast, SAHA stimulation resulted in significant changes expression of 7347 genes strongly affecting signalling pathways, including WNT, NFκB, TGFβ, NOTCH1 and CREB (Additional file 1: Table S1, Fig. 3b).

### ChIP-Seq reveals intergenic regions are subject to change

We mapped changes in histone modification in an attempt to further define the localisation of these changes. Genetic elements such as the transcription start site (TSS) were assessed because of its importance to the regulation of gene expression. To perform this analysis, genomic features were annotated using UCSC Genome Browser data and expressed as the fraction of enrichment of overlapping peaks compared to the remaining intergenic regions. We divided the human genome into the TSS, intron, intergenic region, exon and coding exon as well as CpG Islands (CGI), 5′ and 3′ UTR and the whole gene using Fisher’s exact test. Stimulation with VPA caused a broad distribution of acetylated and deacetylated histones with the majority clearly changed on intronic regions (Fig. 3c). We identify reduced H3K9/14ac is less likely to occur on genes that were upregulated in expression. ChIP-Seq also shows broader changes in H3K4me3 with increases in 5′ UTR, CGI and coding exons (Fig. 3d), whereas the changes observed in H3K9me3 were predominantly intergenic (Fig. 3e).

In contrast, SAHA exposure results in extensive histone deacetylation not limited to the promoter and intergenic regions (Fig. 3f). Mapping of chromatin clearly shows dispersed deacetylation with focal histone acetylation at promoters. Our data revealed histone deacetylation at CpG islands and in close proximity to promoter regions. As expected, increased histone acetylation was positively correlated with intergenic regions. The genomic distribution of H3K4me3 was inversely correlated with H3K9/14ac and consistent with dynamic changes at TSSs, promoter regions, 5′UTRs as well as CGIs (Fig. 3g). We found H3K9me3 enrichment was predominantly intergenic, whereas decreases were observed in exon regions as well as UTRs (Fig. 3h). In summary, while our mapping data show increased histone acetylation at genes, we found dramatic deacetylation of the genome. We also observe H3K4me3 and H3K9me3 are subject to distinct changes following HDAC inhibition, suggesting that they may function collaboratively or independently at target genes.

### Changes in histone modification were weakly correlated with changes in gene expression

**Fig. 4:**
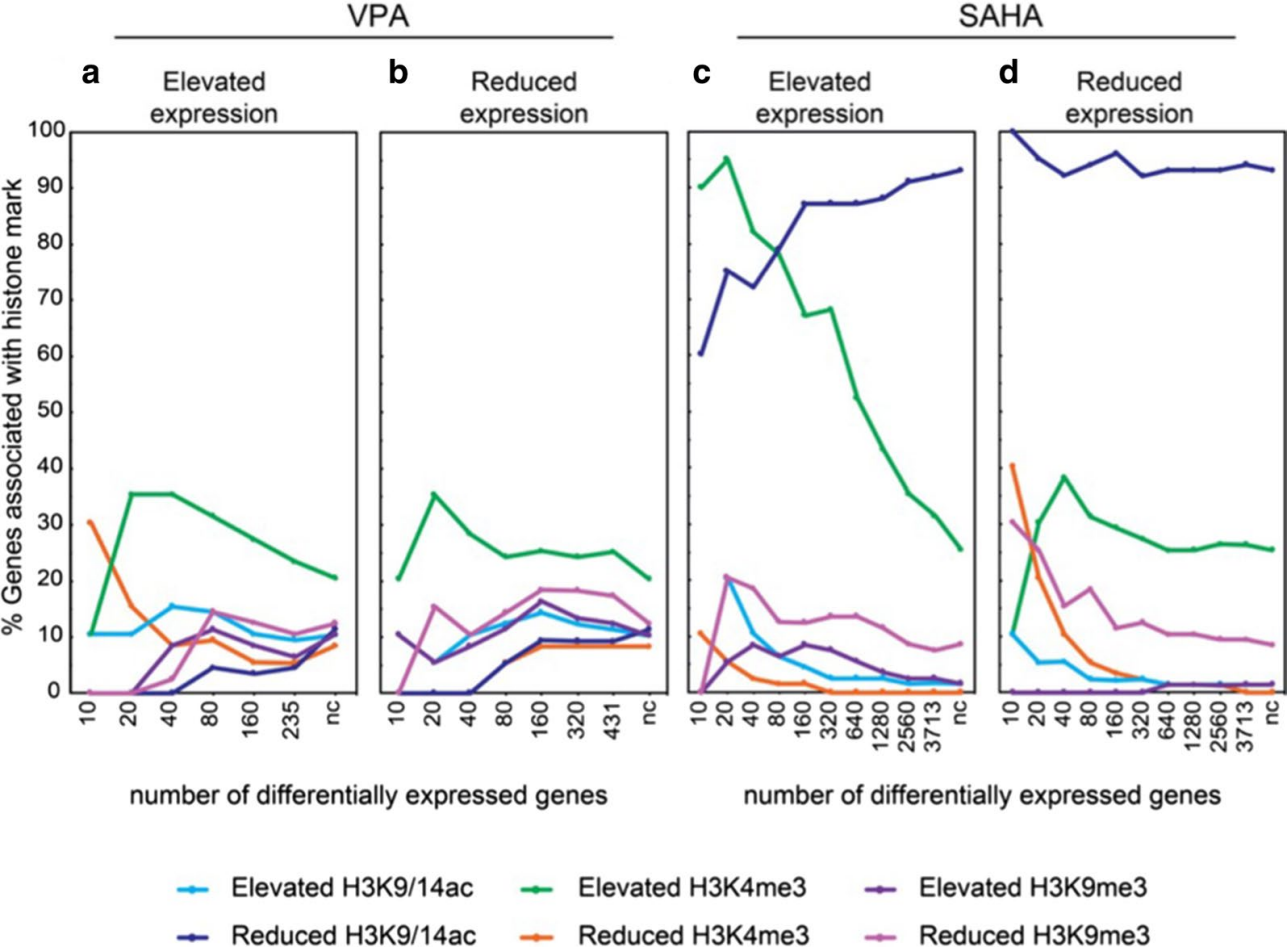
Differential histone modification at the transcription start site associated with gene expression. Correlations are shown for VPA- and SAHA-stimulated cells showing increased (**a**, **c**) and reduced (**b**, **d**) gene expression associated with the histone modifications, H3K9/14ac, H3K4me3 and H3K9me3. Genes are grouped (x-axis) according to increased or reduced expression derived from RNA-seq and shown as a commutative rank (i.e. top 10 genes, top 20 genes, top 40 genes, and so on), with “nc” representing no change (*P* < 0.05). Correlations are shown as the percentage of genes in each rank associated with changes in histone modification within 5-kb window of the transcriptional start site

Next, we determined whether gene expression is associated with histone modification using Fisher’s exact test (Additional file 1: Table S2). In VPA-treated cells, reduced H3K9/14ac was less likely to occur on upregulated genes with a correlation between increased H3K4me3 more likely to occur on downregulated gene targets (Fig. 4a). Reduced H3K9me3 was associated with downregulated genes, whereas increased H3K4me3 was more likely to occur with downregulated expression (Fig. 4b). These results suggest the association between changes in chromatin modification and gene expression is more complicated than previously thought. For example, we did not observe an association for elevated acetylation with gene expression, rather, deacetylation was less likely to occur in upregulated genes (Fig. 4a and b). We did observe an association for elevated H3K4me3 with upregulated gene expression, while no association was observed in downregulated genes (Fig. 4c and d). These observations suggest that while changes in histone modifications in response to VPA or SAHA have some correlation with changes in gene expression, histone acetylation is not the paradigmatic mechanism of transcriptional response following HDAC inhibition.

### VPA and SAHA may alter transcription factor activity and gene expression

Pharmacological HDAC inhibition is believed to alleviate transcriptional suppression by causing histone acetylation and activating gene expression. However, little is known about the specificity of histone deacetylation by VPA and SAHA. We used gene set enrichment analyses (GSEA) to identify transcription factor binding sites (TFBS derived from ENCODE) implicated in histone deacetylation (Additional file 1: Table S3). Our data revealed TFBS that belong to several regulatory factors including NFκB, CTCF, C-JUN and POL-2 are enriched at sites of histone deacetylation following exposure of cells to VPA and SAHA. NFκB, a mediator of inflammation, is strongly implicated in the precipitating development of seizure susceptibility and inflammation in drug-resistant epilepsies [37, 38]. Recent studies have shown HDAC inhibition suppresses pro-inflammatory NFκB target genes in brain injury [39, 40].

### HDAC inhibition influences deacetylation of NFκB pathway genes

**Fig. 5:**
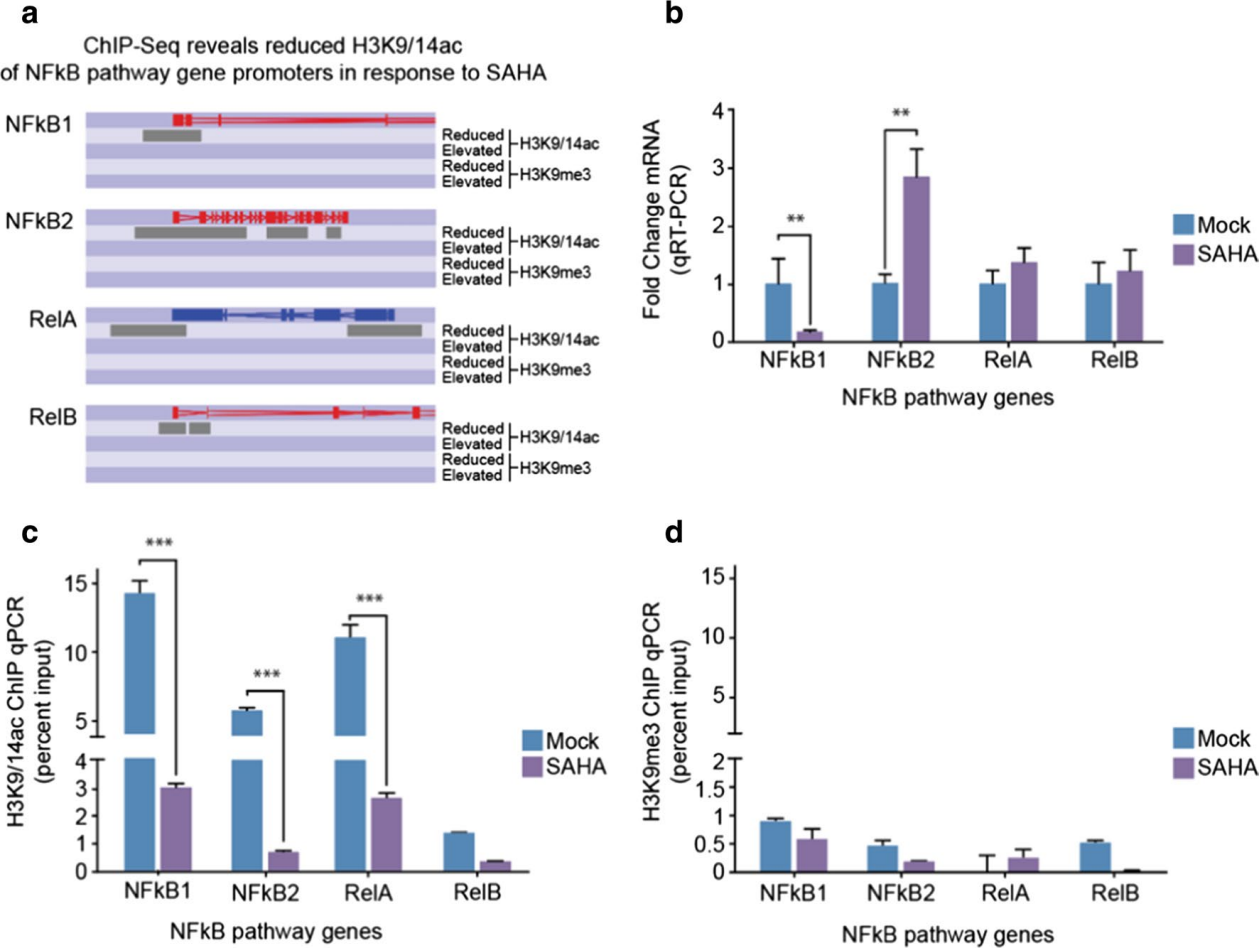
SAHA influences H3K19/14ac and H3K9me3 content on the promoters of NFκB pathway genes as identified by ChIP-Seq. **a** Differential enrichment was determined using MACS and visualised using SeqMonk. **b** Real-time PCR validation of changes in gene expression of NFκB signalling pathway-related genes after exposure to SAHA. Gene expression was analysed in three independent experiments before and after exposure to SAHA. Real-time PCR validation of **c** H3K9/14ac and **d** H3K9me3 by ChIP for NFκB signalling pathway genes in mock and SAHA-exposed cells. Yields were analysed with respect to input. ** = *P* < 0.01 *** = *P* < 0.001

If HDAC inhibition is responsible for H3K9/14ac content than SAHA treatment should alter overall promoter content on target genes. Figure 5a shows H3K9/14ac and H3K9me3 sequencing profiles for NFκB1, NFκB2, RelA and RelB in response to SAHA showing deacetylation of H3K9/14 for NFκB-associated genes. These results illustrate dramatic H3K9/14 deacetylation, whereas H3K9me3 content on NFkB-associated genes was not disrupted by SAHA. We therefore performed independent experiments to assess chromatin modification and gene expression. In cells exposed to SAHA, we observe bidirectional mRNA expression of NFkB1 and NFkB2 target genes using qPCR TaqMan assays (Fig. 5b). Next, we examined histone modification by ChIP. Proteins were cross-linked to DNA and soluble chromatin fractionated by sonication and immunopurified using antibodies that specifically recognise H3K9/14ac and H3K9me3, respectively. The immunopurified DNA was analysed by qPCR using amplimers that are specifically designed to detect NFκB1, NFκB2, RelA and RelB genes. SAHA significantly reduced H3K9/14ac chromatin content on the promoters of the NFκB1, NFκB2 and RelA genes (Fig. 5c). These results show a correspondence between ChIP-Seq and ChIP combined with qPCR signal detection. We also confirmed that SAHA failed to alter H3K9me3 promoter content (ChIP-Seq shown above in Fig. 5a) using highly sensitive ChIP-qPCR (Fig. 5d). The results to these experiments suggest SAHA distinguishes NFκB target genes by regulating promoter content independent of changes in H3K9me3. Taken together, while we observe elevated histone acetylation in response to HDAC inhibitors, we cannot exclude the dominant deacetylation identified using methods designed to fractionate and detect chromatin content.

## Discussion

The perception in the drug development community of restoring gene expression by HDAC inhibition has received considerable attention; however, the mechanism of action mediating chromatin modification and specifically histone deacetylation remains poorly understood [41]. Despite some of the technological advances in mapping, the chromatin field remains largely uncharted with regard to known acetylation and deacetylation gene targets conferred by pharmacological HDAC inhibition. We have performed ChIP-Seq for H3K9/14ac, H3K4me3 and H3K9me3 using soluble chromatin prepared from primary human cells exposed to VPA. The genome-wide distributions that we have performed for histone modifications provide some unexpected results and new clues for understanding how VPA causes chromatinisation events associated with gene activity, which were independently confirmed in experiments with SAHA.

### Clinical perspective

Epilepsy is the most common chronic brain disease. Despite progress in classification and diagnosis including pharmacologic management of seizures, epileptic therapy remains a therapeutic challenge. The pathogenesis of epilepsy involves a complex interaction of genetic and environmental factors and nowhere is this more relevant than the use of epigenetic therapies such as inhibitors that target the HDAC enzymes [24]. Approved by the FDA, SAHA is a drug shown to have neuroprotective effects [42–44]. Preclinical studies have shown benefits of HDAC inhibitors in models of brain injury [45, 46] and intracerebral haemorrhage [47]. For example, VPA and SAHA show therapeutic potential in focal cerebral ischemia [48] and have emerged as key targets to regulate lysine acetylation associated with synaptic plasticity [49]. A role for chromatin modifications influencing dendritic sprouting, synaptic recovery and improved memory was elegantly shown in response to HDAC inhibition [50]. Furthermore, VPA attenuates the redox-sensitive transcription factor NF-κB which is known to influence ischemia-induced signalling in cerebral artery occlusion [51]. While the inhibition of HDAC activity can drive NF-κB gene expression (52), their function is not exclusively activation [53]. Indeed, their effect on inflammatory signalling is dependent on selective NF-κB acetylation sites. This diversity in activation and suppression by pharmacological HDAC inhibition led us to examine more closely the Ac/Dc axis of NF-κB family members.

A key challenge to the therapeutic potential of epigenetic modifiers is explaining how cells exposed to HDAC inhibitors influence distinct deacetylation a response that is considered antithetical. An elegant solution is shown by a recent study examining inflammatory responses [54]. In that study, HDAC3 was shown to precisely regulate transcriptional suppression and activation events when engaged with nuclear receptor corepressors 1 and 2 (NCoR1/2). This dichotomous mechanism involving HDAC activity as an activator and repressor of gene transcription not only challenges the conventional view but raises important questions on the broad role of Ac/Dc axis and the clinical impact of pharmacological HDAC inhibitors [55].

### Histone modification vulnerabilities are associated with gene expression

This study reveals precipitant H3K9/14 deacetylation by VPA that was previously thought to be restricted to cardiac phenotypes [13]. In response to the HDAC inhibitors, H3K4me3 was elevated by SAHA, and this trend was inversely correlated for H3K9me3 by VPA. Our data suggest that this effect paradoxically distinguishes chromatin modification and vulnerabilities by VPA and SAHA. RNA-seq analysis of cells stimulated with VPA shows 520 transcripts differentially regulated and associated with increased H3K4me3 positioned at the transcription start sites (± 5 kb window). This same trend was observed with SAHA altering 7347 transcripts with the majority of activated genes associated with bivalent H3K9/14ac and H3K4me3 marks. In contrast, reduced gene expression was strongly associated with dominant deacetylation of H3K9/14.

Interestingly, NRSF, a transcription factor known to repress transcription of neuronal genes [56], shows discordant association patterns between VPA- and SAHA-exposed cells. This factor is known to exert its repressive function through recruitment of HDAC enzymes. Based on gene set enrichment analysis of the ChIP-seq data, we observe transcription factors such as NRSF are enriched in regions of the genome subject to deacetylation by SAHA, whereas NRSF was enriched in regions of the genome with increased acetylation by VPA. These results show differentially modified regions following VPA and SAHA stimulation did not significantly overlap and likely to be driven by distinct roles by transcription factor activity. Although the logic of chromatin modification by HDAC inhibition is intuitive, this does not exclude other pharmacology. For example, VPA has been shown to cause replication-independent loss of DNA methylation, in addition to, altering histone acetylation content [57].

### Complexity of histone modification identifies pathways of regulation

The major pathways observed with gene expression are consistent with VPA-induced changes in lipid metabolism. For instance, VPA-induced long-chain TAG accumulation, determined by large-scale lipidomic studies, is mediated by fatty acid metabolism [58] and corresponds with lipogenic gene expression changes [59]. Indeed, the selectivity in pathways for VPA was also observed for SAHA and included NFκB and NOTCH signalling. Additionally, the HDAC inhibitor, SAHA, strongly altered signalling pathways mediated by WNT-, NFκB-, TGFβ-, and CREB-binding protein (CREBBP also known as CBP). The impact of HDAC inhibition on the central transcriptional co-activators CBP and P300 suggests a widespread regulatory role conferring signalling robustness.

The traditional observation of HDAC inhibition causing elevated histone acetylation responsible to restore gene expression has been questioned. We have previously described cardiac deacetylation by HDAC inhibition linking CBP and P300 with cognate target genes [12]. Recent evidence from meta-analyses of public datasets shows convergent pathways under control of pharmacological HDAC inhibition. Surprisingly, strong clustering was observed for gene expression irrespective of drug class. For example, isoform-selective HDAC inhibitors such as CHR-3996, largazole, romidepsin and VPA show suppression of EP300 target genes as a prime candidate for regulation as predicted by pan-HDAC inhibitors such as givinostat, ITFA, ITFB, paninoblast, DSAHA and Trichostatin A [15]. Given the strong involvement of CTCF with epigenetic regulatory factors [60] (depending on sequence context this factor can promote both permissive and repressive chromatin structures by recruiting different chromatin modifying complexes), this observation could at least partly explain the broad changes in histone modifications following HDAC inhibition.

### Substrate diversity of lysine deacetylation inhibitors

The conceptual opinion is pharmacological HDAC inhibition functions to restore histone acetylation capacity and thereby regulating gene activation patterns. While this appears to be the case for many genes, our results highlight dramatic deacetylation potentially regulating a large fraction of the transcriptome with other studies highlighting a dichotomous gradient by HDAC activity [54]. Emerging evidence suggests that HDAC inhibition affects the responsiveness of cells to regulate lysine acetylation states. Proteome studies of lysine acetylation reveal substrate and functional diversity of histone and non-histone proteins. Indeed, the scope of non-histone acetylation was shown by peptide immunoaffinity enrichment and mass spectrometry (MS) of proteins outside of the nucleus [61]. High-resolution MS identified 3600 lysine acetylation sites on 1750 proteins involved in cell cycle, transcript splicing and other nuclear functions with surprising specificity to cytoplasmic functions. The striking feature of lysine acetylation is that it targets large macromolecular complexes involved in chromatin remodelling complexes to influence gene expression. Remarkably, inhibition of deacetylase activity using SAHA and MS-275 increased lysine acetylation by approximately 10% with unexpected site specificity. For example, acetylation of histones was not equally increased at all sites revealing these drugs target site activity differently. In addition, there are drug-specific activities with a number of sites on the core histones H3 and H4 that are more highly regulated by SAHA than by MS-275. These results parallel the impact of histone deacetylation by SAHA and VPA observed by ChIP-seq. The functional role of non-histone-regulated lysine acetylation is probably best characterised for the transcriptional co-activators, CBP and P300. Time-resolved acetylome analyses reveal rapid turnover of lysine Ac/Dc to regulate gene expression (62). Quantitative acetylomics identified thousands of in vivo substrates of CBP and P300 that include paradigmatic histone signature sites such as H3 and H4 and new sites such as H2B that are acetylated by the complex and implicated in transcriptional control. This type of global analysis provides a comprehensive view of Ac/Dc dynamics that shape the regulation of protein function (histone and non-histone) to influence gene expression. While there is little doubt that MS-based quantitative mapping is important to understand the functional role of lysine acetylation, a move towards integrating MS mapping with genome-wide patterns such as ChIP-seq will require inclusion of the ENCODE project (https://www.encodeproject.org) to accurately map the distribution of histone acetylation and deacetylation marks together with transcription factor binding to deconvolute gene expression patterns regulated by pharmacological HDAC inhibition.

In conclusion, our genome-wide analyses using ChIP-Seq reveal dramatic acetylation and deacetylation changes for H3K9/14 together with the mapping of sites such as H3K4 and H3K9 methylation represent a first step forward to understanding the actions of VPA and SAHA on the chromatin landscape and a framework for gene expression relevant to clinical epigenetics.

## Supplementary Information

**Additional file 1: Table S1**. Stimulation by VPA and SAHA identifies pathways from GSEA. **Table S2**. Statistical analysis of differential histone modifications associated with gene expression. **Table S3**. Association of TFBS with differential H3K9/14 acetylation

## Data Availability

The datasets analysed in the current study are available from the corresponding author on reasonable request.

## Abbreviations

Ac/Dc: Acetylation/deacetylation index
ChIP: Chromatin immunoprecipitation
ChIP-Seq: Chromatin immunoprecipitation sequencing
CGI: CpG Island
DMEM: Dulbecco's modified Eagle medium
ENCODE: Encyclopaedia of DNA elements
GSEA: Gene Set Enrichment Analysis
H3K9/14ac: Histone H3 Lysine K9/14 acetylation
H3K4me3: Histone H3 Lysine K4 trimethylation
H3K9me3: Histone H3 Lysine K9 trimethylation
HAT: Histone acetyltransferase
HDAC: Histone deacetylase
RNA-seq: RNA sequencing
SAHA: Suberanilohydroxamic acid
Seq: Massive parallel sequencing
TFBS: Transcription factor binding site
TSS: Transcription start site
UCSC: University of California Santa Cruz Genome Browser
UTR: Untranslated region
VPA: Valproic acid
