# Supplementary material for: Epigenetic evidence of an Ac/Dc axis by VPA and SAHA
Source: Clin Epigenetics. 2021 Mar 20;13:58. doi: 10.1186/s13148-021-01050-4 (PMC7981901; doi:10.1186/s13148-021-01050-4)
Supplement: Supplementary file 1 — Additional file 1: Table S1. Stimulation by VPA and SAHA identifies pathways from GSEA. Table S2. Statistical analysis of differential histone modifications associated with gene expression. Table S3. Association of TFBS with differential H3K9/14 acetylation [file 13148_2021_1050_MOESM1_ESM.docx]

**Additional file 1**

Epigenetic evidence of an Ac/Dc axis by VPA and SAHA

Sebastian Lunke^1#^, Scott Maxwell^1,2#^, Ishant Khurana^1,2^, Harikrishnan KN^1,2,4^, Jun Okabe^1,2^, Keith Al-Hasani^1,2^ and Assam El-Osta^1,2,3,4,5,6,7,8^*

^1^Baker Heart and Diabetes Institute, Melbourne, Victoria 3004, Australia, ^2^Epigenetics in Human Health and Disease Laboratory, Central Clinical School, Monash University, Melbourne, Victoria 3004, Australia, ^3^Department of Diabetes, Central Clinical School, Monash University, Melbourne, Victoria 3004, Australia, ^4^Department of Clinical Pathology, The University of Melbourne, Parkville, VIC 3010, Australia, ^5^Department of Medicine and Therapeutics, The Chinese University of Hong Kong, Hong Kong SAR, ^6^Hong Kong Institute of Diabetes and Obesity, Prince of Wales Hospital, The Chinese University of Hong Kong, 3/F Lui Che Woo Clinical Sciences Building, 30-32 Ngan Shing Street, Sha Tin, Hong Kong SAR, ^7^Li Ka Shing Institute of Health Sciences, The Chinese University of Hong Kong, Hong Kong SAR, ^8^University College Copenhagen, Faculty of Health, Department of Technology, Biomedical Laboratory Science, Copenhagen, Denmark. ^#^authors contributed equally.

**Additional file 1**

**Table S1**

**Stimulation by VPA and SAHA identifies pathways from GSEA**

| **SAHA exposed cells** | **P-value** |
| --- | --- |
| Cytoskeleton remodeling_TGF, WNT and cytoskeletal remodeling | 3.04E-10 |
| Apoptosis and survival_HTR1A signaling | 8.62E-09 |
| Development_NOTCH1-mediated pathway for NF-KB activity modulation | 2.28E-08 |
| Apoptosis and survival_BAD phosphorylation | 3.92E-08 |
| Development_TGF-beta receptor signaling | 4.97E-08 |
| Transcription_CREB pathway | 7.23E-08 |
| Cytoskeleton remodeling_Cytoskeleton remodeling | 2.17E-07 |
| Cell adhesion_Chemokines and adhesion | 4.37E-07 |
| Translation_Non-genomic (rapid) action of Androgen Receptor | 5.49E-07 |
| Transcription_Ligand-Dependent Transcription of Retinoid-Target genes | 6.72E-07 |
|  |  |
| **VPA exposed cells** | **P-value** |
| Regulation of lipid metabolism_Regulation of lipid metabolism via LXR, NF-Y, SREBP | 2.66E-06 |
| Regulation of lipid metabolism_Insulin regulation of fatty acid metabolism | 4.28E-05 |
| Development_Role of IL-8 in angiogenesis | 3.67E-04 |
| Cell cycle_Regulation of G1/S transition (part 1) | 6.85E-04 |
| Development_NOTCH-induced EMT | 1.01E-03 |
| Regulation of metabolism_Role of Adiponectin in regulation of metabolism | 1.10E-03 |
| Development_Notch Signaling Pathway | 1.10E-03 |
| Cell cycle_Cell cycle (generic schema) | 1.37E-03 |
| Cytoskeleton remodeling_Regulation of actin cytoskeleton by Rho GTPases | 1.80E-03 |
|  |  |
| **Commonly affected pathways in VPA and SAHA exposed cells** | **P-value** |
| Development_Notch Signaling Pathway | 2.01E-04 |
| Development_NOTCH-induced EMT | 2.74E-04 |
| Cell adhesion_Endothelial cell contacts by non-junctional mechanisms | 5.58E-04 |
| Transport_Macropinocytosis regulation by growth factors | 8.74E-04 |
| Development_Regulation of CDK5 in CNS | 8.85E-04 |
| Cardiac Hypertrophy_NF-AT signaling in Cardiac Hypertrophy | 9.84E-04 |
| Development_NOTCH1-mediated pathway for NF-KB activity modulation | 1.57E-03 |
| Immune response_Oncostatin M signaling via MAPK in human cells | 2.01E-03 |
| Apoptosis and survival_BAD phosphorylation | 2.90E-03 |
| Development_Ligand-independent activation of ESR1 and ESR2 | 3.53E-03 |

**Additional file 1**

**Table S2**

**Statistical analysis of differential histone modifications associated with gene expression**

**VPA exposed cells**

| **Modification** | **p-value** | **Odds ratio** | **Comment** |
| --- | --- | --- | --- |
| **Increased gene expression*** | | | |
| H3K4me3 **↑** | 2.16E-001 | 1.21 | No observed association |
| H3K4me3 **↓** | 1.06E-001 | 0.60 | No observed association |
| H3K9K14ac **↑** | 7.36E-001 | 0.90 | No observed association |
| H3K9K14ac **↓** | 6.21E-004 | 0.37 | Less likely to occur in up-regulated genes |
| H3K9me3 **↑** | 9.65E-002 | 0.63 | No observed association |
| H3K9me3 **↓** | 3.63E-001 | 0.80 | No observed association |
| **Decreased gene expression**** | | | |
| H3K4me3 **↑** | 2.67E-002 | 1.29 | More likely to occur in down-regulated genes |
| H3K4me3 **↓** | 8.58E-001 | 1.03 | No observed association |
| H3K9K14ac **↑** | 2.89E-001 | 1.18 | No observed association |
| H3K9K14ac **↓** | 1.67E-001 | 0.78 | No observed association |
| H3K9me3 **↑** | 2.01E-001 | 1.21 | No observed association |
| H3K9me3 **↓** | 6.57E-003 | 1.44 | More likely to occur in down-regulated genes |

*Odds ratio fold change >1.5, ** Odds ratio fold change <1.5

**SAHA exposed cells**

| **Modification** | **p-value** | **Odds ratio** | **Comment** |
| --- | --- | --- | --- |
| **Increased gene expression*** | | | |
| H3K4me3 **↑** | 7.95E-013 | 1.33 | More likely to occur in up-regulated genes |
| H3K4me3 **↓** | 2.61E-001 | 0.64 | No observed association |
| H3K9K14ac **↑** | 5.96E-002 | 1.37 | No observed association |
| H3K9K14ac **↓** | 2.52E-002 | 0.85 | Less likely to occur in up-regulated genes |
| H3K9me3 **↑** | 6.22E-003 | 1.51 | More likely to occur in up-regulated genes |
| H3K9me3 **↓** | 6.97E-003 | 0.83 | Less likely to occur in up-regulated genes |
| **Decreased gene expression**** | | | |
| H3K4me3 **↑** | 7.78E-001 | 0.96 | No observed association |
| H3K4me3 **↓** | 3.22E-004 | 5.96 | More likely to occur in down-regulated genes |
| H3K9K14ac **↑** | 7.86E-002 | 1.93 | No observed association |
| H3K9K14ac **↓** | 1.66E-001 | 0.78 | No observed association |
| H3K9me3 **↑** | 6.78E-002 | 0.19 | No observed association |
| H3K9me3 **↓** | 3.58E-002 | 1.41 | More likely to occur in down-regulated genes |

*Odds ratio fold change >1.5, ** Odds ratio fold change <1.5

**Additional file 1**

**Table S3**

**Association of TFBS with differential H3K9/14 acetylation**

|  |  |  |  |  |  | | |
| --- | --- | --- | --- | --- | --- | --- | --- |
|  |  | **Transcription Factor** | **Max** ***P-*value** | **Min *P-*value** | | **Max FDR** |  |
| **SAHA** | **Associated with deacetylation** | CTCF | 0.006 | 0.000 | | 0.099 |  |
|  |  | NFKB | 0.020 | 0.000 | | 0.062 |  |
|  |  | C-MYC | 0.002 | 0.000 | | 0.057 |  |
|  |  | NRSF | 0.000 | 0.000 | | 0.051 |  |
|  |  | GR | 0.006 | 0.000 | | 0.043 |  |
|  |  | NRF1 | 0.003 | 0.000 | | 0.072 |  |
|  |  | P300 | 0.000 | 0.000 | | 0.052 |  |
|  |  | C-JUN | 0.046 | 0.001 | | 0.073 |  |
|  |  | POL2(B) | 0.000 | 0.000 | | 0.044 |  |
|  |  | BCL3 | 0.002 | 0.000 | | 0.042 |  |
| **VPA** | **Associated with deacetylation** | POL2 | 0.000 | 0.000 | | 0.004 |  |
|  |  | CTCF | 0.039 | 0.001 | | 0.094 |  |
|  |  | TAF1 | 0.000 | 0.000 | | 0.001 |  |
|  |  | POL2-4H8 | 0.000 | 0.000 | | 0.001 |  |
|  |  | NFKB | 0.019 | 0.000 | | 0.042 |  |
|  |  | NRF1 | 0.005 | 0.000 | | 0.013 |  |
|  |  | STAT3 | 0.000 | 0.000 | | 0.007 |  |
|  |  | TBP | 0.000 | 0.000 | | 0.001 |  |
|  |  | YY1 | 0.003 | 0.000 | | 0.021 |  |
|  |  | C-JUN | 0.043 | 0.001 | | 0.058 |  |
|  | **Associated with acetylation** | NRSF | 0.003 | 0.000 | | 0.000 |  |
|  |  | SUZ12 | 0.024 | 0.000 | | 0.020 |  |
